# Developmental disparities in sedentary time by period of the day among US youth: a cross-sectional study

**DOI:** 10.1186/s12889-022-14447-4

**Published:** 2022-11-08

**Authors:** María Enid Santiago-Rodríguez, Jinsong Chen, Karin A. Pfeiffer, David X. Marquez, Angela Odoms-Young, Eduardo Esteban Bustamante

**Affiliations:** 1 Child Movement, Activity, and Development Health Laboratory, School of Kinesiology (SKB Suite 1000), University of Michigan, 830 N. University Ave, Ann Arbor, MI 48109 USA; 2 College of Applied Health Sciences, University of Illinois Chicago, Chicago, USA; 3 School of Public Health, University of Nevada Reno, Reno, USA; 4 Department of Kinesiology, Michigan State University, East Lansing, USA; 5 Department of Kinesiology & Nutrition, University of Illinois Chicago, Chicago, USA; 6 Division of Nutritional Sciences, Cornell University, Ithaca, USA

**Keywords:** Movement Behavior, Children, Adolescents, Disparities, Accelerometry

## Abstract

**Background:**

Definitive evidence shows sedentary time (ST) is an independent risk factor for chronic disease, irrespective of physical activity. Despite calls to limit youth ST, studies demonstrate a spike in ST at the transition from childhood to adolescence. Identifying periods of the day (e.g., before school, during school, afterschool, and evenings) during which ST is higher in adolescents vs. children—that is, specifying when within daily routines ST disparities emerge—may be important to inform intervention strategies, as periods of the day correspond with variations in setting and supervision. The purpose of this study was to examine device-assessed ST engagement by period of day and developmental stage in a nationally representative sample of United States youth.

**Methods:**

Youth (*N* = 2,972 between 6–18 years) from the 2003–2004 and 2005–2006 waves of NHANES reported demographic variables and wore an accelerometer for seven consecutive days to determine ST. Linear regression analyses were applied to study associations between ST and developmental stage (childhood or adolescence) by period of the week and weekend days, while controlling for sex, race/ethnicity, annual family income, and body mass index.

**Results:**

Adjusted linear regressions (*p*-values < 0.0001) showed that adolescents were more sedentary than children during school, afterschool, and weekday evening periods as well as all the weekend periods. However, during school (36.3 ± 7.3 vs. 28.2 ± 7.2 min/hour; b = -7.4 [-8.1, -6.6]) and afterschool periods (31.1 ± 7.7 vs. 22.7 ± 7.0 min/hour; b = -7.8 [-8.6, -7.0]) showed the largest weekly ST disparities by developmental stage. Overall, the during school and after school hours constitute most (during school = 35% and afterschool = 16%) of the weekly ST disparity between children and adolescents.

**Conclusions:**

Our data provide interventionists with estimates of the potential for ST reduction in each setting and period of the day among US adolescents. Future research should gather information about the barriers and facilitators of ST in adolescents by period of the day to help understand factors driving disparities.

**Supplementary Information:**

The online version contains supplementary material available at 10.1186/s12889-022-14447-4.

## Background

Sedentary time (ST) in children is associated with adverse health risk factors including obesity, poor cardiometabolic markers [1], and poor physical fitness [1, 2]. Addressing ST in youth may be a more effective way of addressing adult chronic disease, rather than waiting until adulthood to treat the issues. Thus, it is important to understand sedentary behavior in youth across stages of development. ST levels begin to rise at age seven and worsen each year with a dramatic increase between 13 and 14 years [3]. This period corresponds with the transition from childhood to adolescence, when new roles, greater independence, and physical maturation coalesce. It has been estimated that US youth, children and adolescents from 6–18 years old [4], spend an estimated 7 h [5] to 9 h per day [6] in ST. ST is substantially higher in adolescents than in children. A study using National Health and Nutrition Examination Survey [7], estimated children between 6 and 11 years old accumulate approximately 6 h per day of ST; this increases to nearly 8 h per day in 12–15 year-olds, and 8.5 h per day in 16 to18 years olds. Although there have increasingly been calls to limit ST [8], the data suggest major development transitions correspond with increases in ST and these increases are unwavering.

Youth showed lower levels of ST during weekdays compared to weekend days over a two-year period in 970 youth (10 to 13 years of age) in Finland [9]. One potentially vital insight is an understanding of the periods of the day during which ST is accumulating in children and adolescents as well as where disparities are highest between adolescents and children. This information may be helpful for determining when (i.e., before school, during school, afterschool, and evening) and on which type of day (weekday vs weekend day) adolescents are more sedentary. Such information would allow interventionists to target periods of the day with the most potential for improvement. To our knowledge, three studies have evaluated ST patterns in children and adolescents by period of the day; one in Belgium, one in Spain, and one in Norway [10–12].

In Belgium, investigators examined the frequency of device-based ST bouts in children (*N* = 740, mean age = 10.9 years) of various durations (i.e., 5–10 min, 10–20 min, and 30 + minutes) during each period of the weekday (i.e., before school, during school, afterschool, and evening) [10]. In other words, when researchers assessed the frequency of ST in 10-min bouts during school hours, they captured the number of times a child remained sedentary for 10 consecutive minutes during school hours. The authors found that—regardless of bout duration—children engaged in more sedentary bouts during evening hours (6:00–10:00 p.m.) than school hours (8:30 a.m. – 4:00 p.m.), and more sedentary bouts during school hours than after-school hours (4:00 – 6:00 p.m.). In this case, researchers operationalized ST in a way that is difficult to translate into minutes of ST. Minutes of ST is the most intuitive framing for parents, teachers, and children seeking to reduce ST. Lastly, although the researchers defined the periods of the day, they did not take into account before school hours, did not compare weekdays vs weekend days, nor did they include adolescents.

In Spain, researchers studied disparities in ST between school hours (e.g. recess, physical education class, and lunchtime) and out of school hours [11]. They recruited 826 children attending 1^st^ to 4^th^ grades and 678 adolescents attending 7^th^ to 10^th^ grades to measure ST with accelerometers over a three-year period. Their findings revealed adolescents were more sedentary than children during school hours, out of school hours, during recess, during physical education class, and during the weekend. In this case, authors reported out of school time and weekend time as single values, rather than breaking them down into their constituent parts. This makes it hard to determine the extent to which out of school ST was driven by before school time, afterschool time, or evening hours, which limits our ability to inform interventions to maximize ST reductions.

Finally, in Norway, a large nationally representative cross-sectional study [12] tested the changes in ST from 2005 to 2018 in children (9-year-old) and adolescents (15-year-old) by periods of the day (before school, during school, and afterschool). They reported there was a significant increase in ST afterschool in 9-year-old boys from 2005 to 2018, but not in the morning or during school. Also, there was a significant increase in ST during school from 2005 to 2018 among 15-year-old boys, but not in the morning or afterschool time. Findings suggest ST is higher in adolescents than children but some limitations remain. First, authors determined changes in ST across the period of the day within each developmental stage but did not test the difference in ST across developmental stages. Second, ST at age nine may not be representative of ST throughout childhood and ST at age 15 may not represent ST behavior across adolescence. Lastly, authors did not breakdown the weekend day into periods, precluding determination of when ST disparities by developmental stage take place on weekends.

Despite the body of knowledge about ST differences by developmental stage, some gaps in knowledge remain. First, previous studies were conducted in Belgium, Spain, and Norway but none have been conducted in the US. Second, none of the studies used a nationally representative sample including a broad age range; limiting the generalizability of ST comparisons by developmental stage. Third, no study broke down weekend time into component parts. We contend that addressing these gaps will provide an understanding of children’s ST patterns that will inform interventions by elucidating when youth developmental stage disparities emerge and how much potential each period of the day has for addressing these disparities. These are important insights given periods of the day correspond with variations in youth location, activities, and adult supervision.

Hence, the purpose of this study was to examine device-assessed ST engagement by periods of day in a nationally representative sample of US youth. Our first aim was to determine the periods of the weekday (before school, during school, afterschool, and evening) during which adolescents were more sedentary than children. We hypothesized children (ages 6–12 years) would be less sedentary during school and afterschool hours compared to adolescents (ages 13–18 years)—but not before school or during the evening—on an average weekday. Our second aim was to determine the times of day (morning, afternoon, and evening) during which adolescents were more sedentary than children during an average weekend day. It was hypothesized that children would be less sedentary during the afternoon and evening compared to adolescents—but not in the morning—on an average weekend day. Our hypotheses are based on data showing adolescents are more sedentary than children when evaluating the entire day [3] regardless of a weekday or weekend day [12]; and that adolescents are more sedentary than children during particular periods within the school day such as recess and lunchtime [12]. Similarly, previous studies have reported children are less sedentary during afterschool hours compared to during school hours [11]. Although no previous study has provided data about the before school period, we do not anticipate any difference since children and adolescents are expected to have similar routines before school (e.g., having breakfast, getting dressed, brushing their teeth).

## Methods

This study is a secondary analysis of cross-sectional National Health and Nutrition Examination Survey (NHANES) data aiming to test developmental stage disparities by periods of the weekday and weekend day.

### National Health and Nutrition Examination Survey

The NHANES has been described in detail elsewhere [13]. Briefly, this nationally representative study examines roughly 5,000 participants on a host of health-related outcomes annually. Thus, participants are different in each wave. New cohorts are brought in each year and tested using a complex sampling design to ensure national representation. NHANES evaluates physical health and lifestyle behaviors (e.g. nutrition, physical activity, and screen time) in US children and adults through an interview and a physical examination. The interview provides data related to demographics, socioeconomic status, and lifestyle behaviors (e.g. nutrition, physical activity, screen time); while the physical examination provides data about laboratory tests, medical, dental, and physiological information. During the years 2003–2006, NHANES also collected accelerometer data on 5,546 youth.

### Participants

**Fig. 1:**
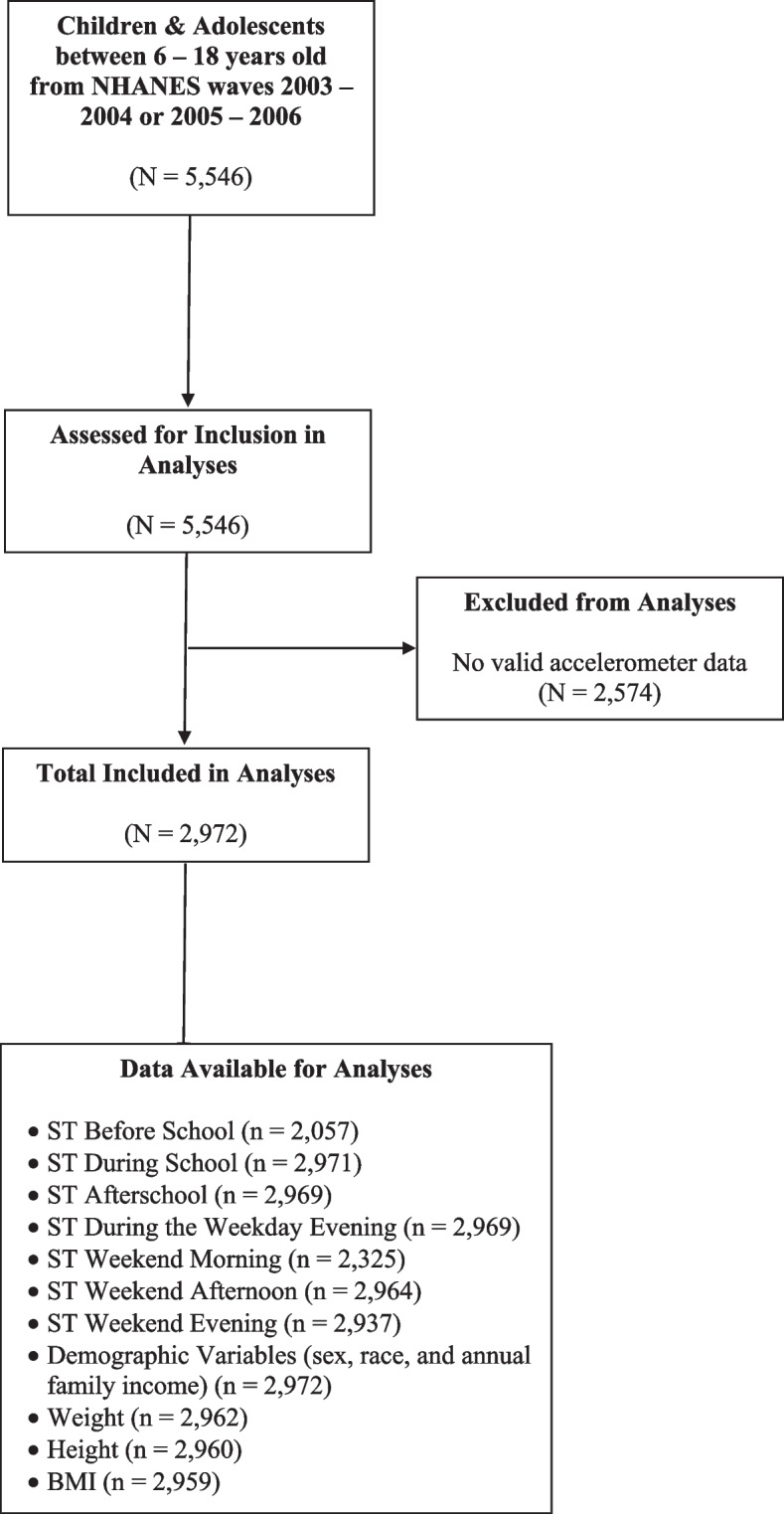
Participants Sample Size Flow Diagram

The sample for this study consisted of youth between 6 to 18 years old from the 2003 – 2004 and 2005 – 2006 waves of the NHANES. The final sample consisted of 2,972 children and adolescents. See Fig. 1 for more details about how we obtained the final sample. Sample size calculations were based on Green’s formulas [14], which showed that for one model with covariates, a total of 199 participants were needed; however, after adjusting for multiple calculations (a total of 7 regressions), then a sample size of 1,393 was needed to achieve a statistical power of 80% using a family-wise significance level of 0.05 (0.05/7 for each model) to test moderation effects for the entire model and its coefficients. Thus, 0.05/7 = 0.07 and any *p* < 0.007 will be considered significant.

### Variables

#### Sedentary Time (ST)

ST was obtained via accelerometry. Participants were instructed to wear an ActiGraph 7164 accelerometer (ActiGraph, Pensacola, FL) for seven consecutive days attached to their right hip with an elastic band. To obtain hour-by-hour ST (minutes/hour) during an average weekday and weekend day, the data were analyzed using the Web App for Processing NHANES Accelerometer Data [15]. The Web App is an R package containing the NHANES data and functions by processing the data depending on the selected features. The App provides the option to export a processed data file into R, SAS, STATA, or SPSS (see Table A1, which summarizes the features selected to obtain the hour-by-hour ST data from the Web App). After obtaining the hour-by-hour ST, average minutes/hour of ST was calculated for each period of the day for each participant. For weekdays, the periods of the day corresponded to before school (between 6:00 a.m. and 7:59 a.m.), during school (between 8:00 a.m. and 2:59 p.m.), afterschool (between 3:00 p.m. and 5:59 p.m.), and evening at home (between 6:00 p.m. and 9:00 p.m.). The breakdown of the weekday periods was based on most US public school schedules [16]. For the weekend day the periods of the day corresponded to morning (between 7:00 a.m. and 11:59 a.m.), afternoon (12:00 p.m. and 5:59 p.m.), and evening (6:00 p.m. and 10:00 p.m.). These were based on the weather forecast [17] and the US average waking time [18] and bedtime [19].

#### Demographic & health-related variables

Age, annual family income, and sex were obtained from the demographic data reported via interview. Age was reported in years; however, a categorical variable was created called developmental stage, which was defined as childhood (6–12 years old) and adolescence (13–18 years old). Annual family income was defined as the total family income during the wave of data collection. Participants selected an income range that spanned between $0 and $75,000 + in $5,000 increments. However, we grouped the participants’ answer into a dichotomous variable: under $20,000 and 1: $20,000 and over. For contextual purposes, $20,000 in 2006 is equivalent to $29,392.46 in 2022 based on the CPI Inflation Calculator [20]. Body mass index (kg/$${m}^{2}$$) was obtained from height and weight collected via the NHANES body measurement protocol.

### Statistical analyses

Frequencies and descriptive statistics were conducted for all the demographic variables as well as ST for each period of the day during weekday and weekend day. All analyses were conducted using SAS version 9.4. A significance level of 0.05 was used.

To test hypothesis 1, linear regression analyses were conducted separately for each of the four outcomes: ST before school, ST during school, ST afterschool, and ST during the evening. To test hypothesis 2, the above-described linear regression analyses were conducted separately for each of the three outcomes: ST during the morning, ST during the afternoon, and ST during the evening (see Table A2, which provides more details about the linear regression syntax example in SAS that incorporates NHANES complex sampling design to account for stratification, clustering, and weighting). In each linear regression, the predictor variable was developmental stage (1: children, 0: adolescent). Sex, race/ethnicity, annual family income, and body mass index were covariates in the model.

Sensitivity analyses were conducted to ensure that the established timeframes for each period of the day did not result in a difference in ST. Thus, the same linear regression analyses were conducted shifting one hour earlier and later. Therefore, for the one-hour earlier shift, the periods were: before school (between 5:00 a.m. and 6:59 a.m.), during school (7:00 a.m. to 1:59 p.m.), afterschool (2:00 p.m. to 4:59 p.m.), weekday evening (5:00 p.m. to 8:00 p.m.), weekend morning (6:00 a.m. to 10:59 a.m.), weekend afternoon (11:00 a.m. to 4:59 p.m.), and weekend evening (5:00 p.m. to 9:00 p.m.). For the one hour later shift, the periods were: before school (7:00 a.m. to 8:59 a.m.), during school (9:00 a.m. to 3:59 p.m.), afterschool (4:00 p.m. to 6:59 p.m.), weekday evening (7:00 p.m. to 10:00 p.m.), weekend morning (8:00 a.m. to 12:59 p.m.), weekend afternoon (1:00 p.m. to 6:59 p.m.), and weekend evening (7:00 p.m. to 11:00 p.m.).

Assumptions, including normality, linearity, multicollinearity, autocorrelation, and homoscedasticity were examined. Variables that exceeded a kurtosis of ± 3 or skewness of ± 1 were transformed to achieve normality [21]. Residual plots were created to detect outliers and verify if the relationship between the dependent and independent variables was linear [22]. The Variance Inflation Factor (VIF) was used to test for multicollinearity. The parameter to determine the absence of multicollinearity within the variables included in the linear regression was a VIF < 100 [23]. To determine the presence of autocorrelation, a d-value from the Durbin-Watson’s d test between 1.5 and 2.5 was considered as no presence of autocorrelation [23]. Finally, partial correlation plots were used to detect homoscedasticity. These allowed us to check if the residuals were equal across the regression line. Where this held true, this assumption was met [23].

## Results

The study sample consisted of 2,972 children and adolescents with a mean age of 12.5 ± 3.6 years and a mean body mass index of 21.5 ± 5.7 kg/m^2^. Overall, youth spent an average of 474.6 ± 149.0 min/day (≈ 7.9 h/day) in ST. Children’s overall ST was 408.8 ± 127.1 min/day (≈ 6.8 h/day) and adolescents’ overall ST was 533.6 ± 142.4 min/day (≈ 8.9 h/day). Table 1 provides descriptive characteristics of the sample partitioned by developmental stage. Body mass index was transformed using the natural logarithm function. All other assumptions were met.

**Table 1:** Descriptive characteristics by developmental stage

	**N**	**Childhood (C)**	**Adolescence (A)**
Age (years) Mean ± SD	C: 1405, A: 1567	9.2 ± 2.1	15.3 ± 1.7
Overall ST (min/day) Mean ± SD	C: 1405, A: 1567	408.8 ± 127.1	533.6 ± 142.4
BMI (kg/$${m}^{2}$$) Mean ± SD	C: 1400, A: 1559	19.1 ± 4.6	23.7 ± 5.8
Sex			
Female (n)		699	786
Male (n)		706	781
Annual Family Income			
Under $20,000 (n)		348	417
$20,000 and over (n)		1026	1107
Refused or Don’t Know (n)		12	31

*SD* Standard deviation, *BMI* Body mass index

Sensitivity analyses showed that regression models remained significant when shifting the time frames one hour earlier and later for all periods of the day besides before school. The unadjusted model including ST before school with a shift of one hour earlier (5:00 a.m. to 6:59 a.m.) showed developmental stage was not a significant predictor. This was not a concern since no difference in this earlier hour shift may reflect time in which youth were sleeping, which is not considered ST. Thus, it was decided to define the before school period as the time between 6:00 a.m. and 7:59 a.m., as originally planned.

### Hypothesis 1 results: ST by periods of an average weekday

**Fig. 2:**
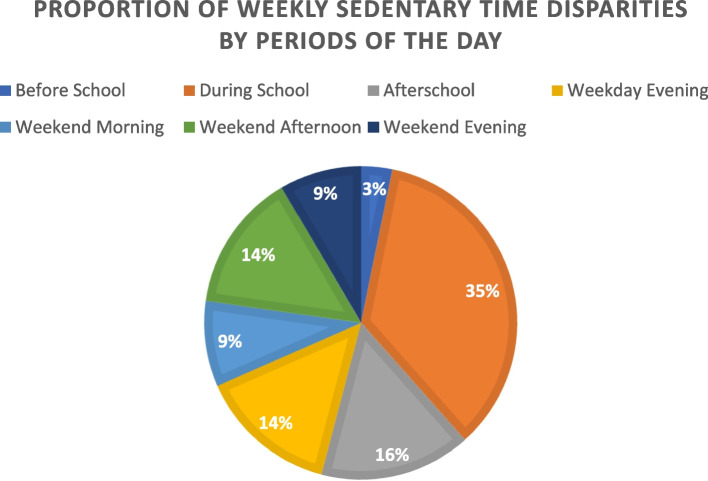
Proportion of Sedentary Time Disparities that each Period of the Day Accounts for an Average Week **Note.** To determine the percentage of sedentary time disparity for each period of the day, the weekly differences (min/week) from Tables 2 and 3 were used. First, we calculated the total sum of the weekly differences for each period of the day (weekday and weekend periods). Secondly, we divided each weekly difference by the total sum and multiplied by 100 to obtain the percentages

**Fig. 3:**
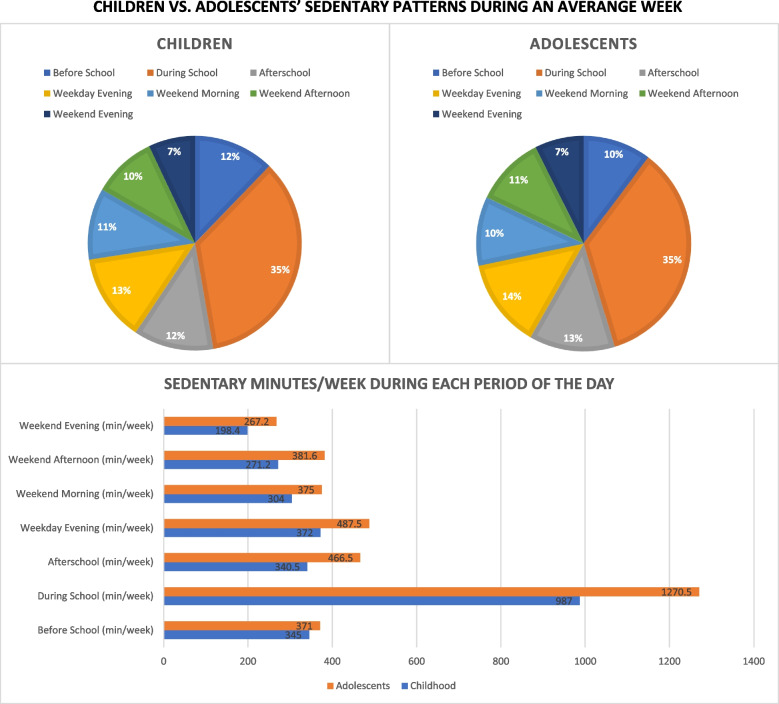
Sedentary Patterns for each Period of an Average Week by Developmental Stage **Note.** To determine the percentage of sedentary time for each period of the day, the average sedentary time in min/hour for each period of the day and developmental stage from Tables 2 and 3 were used. First, we converted min/hour into min/day by multiplying min/hour in a particular period by the length of the period in a given day (before school: 2 h, during school: 7 h, afterschool and weekday evening: 3 h, weekend morning: 5 h, afternoon: 6 h, and evening: 4 h). Then, we multiplied the min/hour by the number of days to which each period corresponds (all weekday periods were multiplied by 5 and all weekend periods were multiplied by 2). Then, we summed these averages to calculate total weekly sedentary time (visible in bar graph below), divided the individual period of the day by the weekly sum, and multiplied by 100. For example, to determine the proportion of weekly sedentary time accounted for by weekday mornings in children, we summed weekly sedentary time to 2,818 min and divided 345 by 2,818 = .07; .07 * 100 = 7%

Table 2 illustrates children were significantly less sedentary than adolescents in all periods of an average weekday besides before school. Also, it presents details about the magnitude of ST difference by developmental stage during an average weekday by day, week, and month. Figure 2 visually demonstrates the proportion of ST disparities accounted for by each period of the day during an average week. More than half of the weekly disparity consists of just two periods of the week: during school and after school. Figure 3 presents the proportion of ST accounted for and the estimated weekly minutes accumulated during each period of the day in children and adolescents, separately. Here, ST contributions are proportional to the time spent in each setting regardless of developmental stage. Slightly less than half of total weekly ST in youth is accumulated during school and after-school periods regardless of developmental stage.

**Table 2:** Patterns of sedentary time during an average weekday by developmental stage

	**N**	**Childhood (C)**	**Adolescence (A)**	**C vs A Differences**
Weekday (Mean ± SD)				
ST Before School (min/hr)	C: 942, A: 1115	34.5 ± 13.8	37.1 ± 13.0	2.6
ST During School (min/hr)	C: 1405, A: 1566	28.2 ± 7.2	36.3 ± 7.3	8.1*
ST Afterschool (min/hr)	C: 1403, A: 1566	22.7 ± 7.0	31.1 ± 7.7	8.4*
ST during Evening (min/hr)	C: 1404, A: 1565	24.8 ± 7.9	32.5 ± 8.2	7.7*
	**C vs A Differences (min/hr)**	Daily Differences (min/day)	Weekly Differences (min/week)	Monthly Differences (hr/month)
Extrapolated Differences in ST				
ST Before School	2.6	5.2	26	1.7
ST During School	8.1*	56.7	283.5	18.9
ST Afterschool	8.4*	25.2	126.0	8.4
ST during Evening	7.7*	23.1	115.5	7.7

*ST* Sedentary time, *SD* Standard deviation, ST Before School = 6 – 7:59 AM, ST During School = 8 AM – 2:59 PM, ST Afterschool = 3 – 5:59 PM, ST During Evening Week Day = 6 – 9 PM, *p < 0.007. To extrapolate differences in ST, the children vs adolescents difference was multiplied by the length of the period in a given day (before school: 2 h, during school: 7 h, afterschool and evening: 3 h) to obtain the daily difference. Then, the daily difference was multiplied by 5-days since youth attend school from Monday to Friday to obtain the weekly difference. Next, the weekly difference was multiplied by four assuming that a given month has 4-weeks to obtain the monthly difference

The unadjusted model indicated that developmental stage was not a significant predictor of ST before school (*F*(1, 30) = 6.8, *p* = 0.01; b = -2.7 [-4.8, -0.6]) (see Table A3). ST data during school showed a difference of 8.1 min/hour by developmental stage. Over the course of a month, these differences constitute a 19-h ST disparity between children and adolescents. In all, the during school period accounted for 35.2% of the weekly disparity in ST between children and adolescents The unadjusted model showed that developmental stage was a significant predictor of ST during school (*F*(1, 30) = 587.5, *p* < 0.0001; b = -8.2 [-8.9, -7.5]). During school ST remained significant in the adjusted model (*F*(8, 30) = 118.5, *p* < 0.0001; b = -7.4 [-8.1, -6.6]). This implies that children were less sedentary during school compared to adolescents after adjusting for sex, race/ethnicity, annual family income, and body mass index. Significant covariates of ST during school (see Table A3) included sex (b = 2.8, *p* < 0.0001) and body mass index (b = 3.8, *p* < 0.0001).

A difference of ST afterschool of 8.4 min/hour was observed when comparing children and adolescents. Over the course of a month, these differences constitute an 8-h ST disparity between children and adolescents. In all, the afterschool period accounted for 15.6% of the weekly disparity in ST between children and adolescents. The unadjusted model showed that developmental stage was a significant predictor of afterschool ST (*F*(1, 30) = 580.8, *p* < 0.0001; b = -8.9 [-9.7, -8.2]). Afterschool ST remained significant in the adjusted model (*F*(8, 30) = 132.2, *p* < 0.0001; b = -7.8 [-8.5, -7.0]), which means that being a child was associated with less ST after school compared to being an adolescent even after including covariates in the model. Significant covariates of ST afterschool (see Table A3) included body mass index (b = 5.1, *p* < 0.0001) as well as being female (b = 2.1, *p* < 0.0001) and Non-Hispanic Black (b = -1.2, *p* = 0.003).

ST data during the weekday evening showed a difference of 7.7 min/hour when comparing children to adolescents. Over the course of a month, these differences also constitute an 8-h ST disparity between children and adolescents. In all, the evening period accounted for 14.4% of the weekly disparity in ST between children and adolescents. The unadjusted model showed that developmental stage was a significant predictor of ST during the evening (*F*(1, 30) = 702.7, *p* < 0.0001; b = -8.4 [-8.1, -7.2]) and this remained significant after adjustment for covariates (*F*(8, 30) = 106.7, *p* < 0.0001; b = -7.6 [-8.5, -6.8]). This implies children accumulated less ST during the evening compared to adolescents, after adjusting for sex, race/ethnicity, annual family income, and body mass index. Significant covariates of ST during the evening (see Table A3) included being female (b = 1.4, *p* = 0.004) and Non-Hispanic Black (b = -2.0, *p* = 0.004).

### Hypothesis 2 results: ST by periods of an average weekend day

Table 3 presents that children were less sedentary compared to adolescents in all periods of an average weekend day. Disparities were highest during the weekend afternoon period. Table 3 also presents details about the differences in ST for each period during an average weekend day by developmental stage and extrapolations for differences per day, week, and month. Figure 2 visually demonstrates the proportion of ST disparities accounted for by each period of the weekend day during an average week; most of the weekend disparity happens during the afternoon period. Figure 3 presents the proportion of ST accounted for each period of the day and estimated weekly minutes of ST by each period of the day in children and adolescents, separately. Again, ST accumulation by period of the day was proportional to the duration of each period, regardless of developmental stage. In both cases, 75% of weekend ST occurred during the morning and afternoon hours.

**Table 3:** Patterns of Sedentary Time During an Average Weekend Day by Developmental Stage

	**N**	**Childhood (C)**	**Adolescence (A)**	**C vs A Differences**
Weekend Day (Mean ± SD)				
ST During Morning (min/hr)	C: 1185, A: 1140	30.4 ± 13.1	37.5 ± 13.2	7.1*
ST During Afternoon (min/hr)	C: 1399, A: 1565	22.6 ± 8.6	31.8 ± 9.0	9.2*
ST during Evening (min/hr)	C: 1393, A: 1544	24.8 ± 10.0	33.4 ± 9.8	8.5*
	**C vs A Differences (min/hr)**	Daily Differences (min/day)	Weekly Differences (min/week)	Monthly Differences (hr/month)
Extrapolated Differences in ST				
ST During Morning	7.1*	35.5	71.0	4.7
ST During Afternoon	9.2*	57.6	115.2	7.7
ST during Evening	8.5*	34.0	68.0	4.5

*ST* Sedentary time, *SD* Standard deviation, ST During Morning = 7 AM – 11:59 AM, ST During Afternoon = 12 PM – 5:59 PM, ST During Evening Weekend Day = 6 PM – 10 PM, *p < 0.001. To extrapolate differences in ST, the children vs adolescents difference was multiplied by the length of the period in a given day (morning: 5 h, afternoon: 6 h and evening: 4 h) to obtain the daily difference. Then, the daily difference was multiplied by 2-days since the weekend was defined as the days in which youth did not attended school (Saturday and Sunday) to obtain the weekly difference. Next, the weekly difference was multiplied by four assuming that a given month has 4-weeks to obtain the monthly difference

Weekend morning ST data revealed a difference of 7.1 min/hour when comparing children to adolescents. Over the course of a month, these differences also constitute a 5-h ST disparity between children and adolescents. In all, the weekend morning period accounted for 8.8% of the weekly disparity in ST between children and adolescents. The adjusted model controlled for sex, race/ethnicity, annual family income, and body mass index. The unadjusted models indicated that developmental stage was a significant predictor of ST during the morning (*F*(1, 30) = 65.0, *p* < 0.0001; b = -7.7 [-9.6, -5.7]). Developmental stage remained a significant predictor of ST during the weekend morning (*F*(8, 30) = 24.6, *p* < 0.0001; b = -7.1 [-9.0, -5.1]) after including covariates in the model. Therefore, being a child was associated with less morning ST compared to adolescents after adjusting for sex, race/ethnicity, annual family income, and body mass index. Significant covariates of ST during the morning (see Table A4) were identifying as Non-Hispanic Black (b = 4.0, *p* < 0.0001) and Other Hispanic (b = 4.5, *p* = 0.0005).

Weekend afternoon ST data revealed a difference of 9.2 min/hour when comparing children to adolescents. Over the course of a month, these differences also constitute an 8-h ST disparity between children and adolescents. In all, the weekend afternoon period accounted for 14.4% of the weekly disparity in ST between children and adolescents. The unadjusted models indicated that developmental stage was a significant predictor of ST during the afternoon (*F*(1, 30) = 480.8, *p* < 0.0001; b = -10.1 [-11.0, -9.1]). Developmental stage remained a significant predictor of ST during the afternoon (*F*(8, 30) = 92.3, *p* < 0.0001; b = -8.9 [-9.9, -7.9]) after including covariates in the model. Therefore, being a child was associated with less afternoon ST, compared to being an adolescent, after adjusting for sex, race/ethnicity, annual family income, and body mass index. Being a female (b = 1.7, *p* = 0.0001), and body mass index (b = 5.5, *p* < 0.0001) were significant covariates of ST during the afternoon (see Table A4).

Finally, weekend evening ST data revealed a difference of 8.5 min/hour when comparing children to adolescents. Over the course of a month, these differences also constitute a 5-h ST disparity between children and adolescents. In all, the weekend evening period accounted for 8.4% of the weekly disparity in ST between children and adolescents. The unadjusted models indicated that developmental stage was a significant predictor of ST during the evening (*F*(1, 30) = 310.2, *p* < 0.0001; b = -9.1 [-10.2, -8.1]). Developmental stage remained a significant predictor of ST during the evening (*F*(8, 30) = 60.5, *p* < 0.0001; b = -8.1 [-9.3, -7.0]) after including covariates in the model. Therefore, being a child was associated with less evening ST compared to being an adolescent after adjusting for sex, race/ethnicity, annual family income, and body mass index. Body mass index (b = 5.0, *p* < 0.0001) was a significant covariate of ST during the evening (see Table A4).

## Discussion

Findings from this study provide insight about the periods of day during which adolescents are more sedentary than children during the week and weekend using a representative sample of US youth between six and 18 years old. Overall, adolescents were more sedentary than children in all periods besides before school. For children (mean ST = 408.8 min/day ≈ 6.8 h/day) and adolescents (mean ST = 533.6 min/day ≈ 8.9 h/day), similar levels of overall ST were observed when compared to previous ST data in US youth which reported that children spend up to 6.0 h/day and adolescents spend up to 8.5 h/day in ST [3].

Within a weekday, the afterschool period showed the largest difference in ST (8.4 min/hour) by developmental stage. However, when the differences at each weekday period were extrapolated, the during school period emerged as the most potentially promising time to reduce ST developmental stage disparities since the daily difference between children and adolescents almost reached one hour (56.7 min/day or 35% difference). On the other hand, the weekend day data by developmental stage revealed that the smallest difference in ST occurred during the weekend morning period (7.1 min/hour) and the largest difference occurred during the weekend afternoon period (9.2 min/hour). Similarly, when the differences at each weekend day period were extrapolated, the afternoon period emerged as the most potentially promising time to reduce these disparities since the daily difference between childhood and adolescence was almost an hour (57.6 min/day or 14% difference). Previous research has shown that if youth substitute 60 min of ST with 60 min of light physical activity [24], moderate to vigorous physical activity [25], or 60 min of sleep [24], it can result in improved body composition [25] and mood [24]. Hence, addressing during school ST disparities (or weekend afternoon ST disparities) alone would substantially improve adolescent health.

The accelerometer recorded ST disparities by developmental stage were much larger on weekdays afterschool and during weekend afternoon periods. This may reflect a shift in structured programming between childhood and adolescence. Adolescents are much less likely to be enrolled in structured extracurricular physical activity programs. In adolescence, academic demands also increase and homework responsibilities may displace the physical activity that adolescents tend to participate in during the afternoon hours when they are younger.

Evidence supports the potential role of increasing schoolwork demands on ST as children age [26]. A Canadian qualitative study conducted a survey with open-ended questions among 102 fulltime undergraduate students and found that attending classes as well as studying for classes at home was their main barrier to reduce ST [26]. Although the current study included few adolescents that could attend college (mainly those between 17 and 18 years of age), the transition from primary school to secondary school also results in greater schoolwork demands.

Our weekday findings provided evidence to support our first hypothesis that children would be less sedentary than adolescents during school and afterschool periods. However, it was surprising that developmental stage was a significant predictor of ST during the evening. In terms of the weekday evening difference in ST, the first hours (6:00 p.m. – 7:30 p.m.) of the weekday evening period can be an extension of the afterschool period, in which some youth are still participating in afterschool activities, though perhaps, accessibility to screens and homework demands also play a role here. Similarly, weekend data supported our second hypothesis that children would be less sedentary during the afternoon and evening compared to adolescents. However, the differences during the weekend morning period were unexpected and can be explained, in part, by adolescents’ accessibility to electronic devices that promote ST compared to children’s accessibility to such devices. For instance, some parents ask children to make their bed, take a shower, and have breakfast before starting the day, and if screen time is part of the routine, it is probable that they will engage in screen time under the adult’s supervision.

Our findings confirm previous literature stating that adolescents are more sedentary than children during the weekday periods and weekend. In Spain, a cross-sectional study revealed male adolescents spend 255.6 ± 32.3 min/day sedentary compared to male children who spent 192.7 ± 27.4 min/day [11]. A similar trend was reported by the authors for female adolescents (275.3 ± 35.6 min/day) and children (201.4 ± 27.6 min/day). The same authors reported children were less sedentary than adolescents during the weekend; here again, our US sample findings are similar to the Spanish sample. However, our findings provide further information by breaking down the prolonged periods into shorter periods that coincide with shifts in setting, activity, and supervision. In the long run (weekly and monthly differences), targeting ST during school and weekend afternoons, present the most profound opportunity to close the ST gap by developmental stage. Although previous interventions have included ST components, achieved health benefits, and been proven to reduce ST (mainly screen time) [27, 28], none have targeted specific periods of the day. The current study suggests future interventions on ST in adolescence should focus on the periods of the day with greater opportunity to help reduce ST disparities by developmental stage such as during school, afterschool, and weekend afternoon periods.

It is believed that recreational screen time is a likely contributor to disparities in ST between adolescents and children. With age, parents give children more autonomy and discretion to decide how they use their leisure time. This can promote the use of smartphones, desktop, and laptop computers, which are very accessible to adolescents. Since 2014 – 2015, there has been a 22% increase (from 77 to 95%) in the number of US adolescents who have access to a smartphone and a 3% increase (from 87 to 90%) in those who have access to desktop and laptop computers [29]. Furthermore, accessibility to these devices can be problematic because adolescents are likely to spend more time in passive activities (e.g., shows, movies, gaming, social media), than cognitively demanding enriching activities (e.g., reading books, playing chess, doing math problems). Most passive activities are designed to grab our attention and not let it go, using features such as never-ending scroll, streaks, and notifications which can result in developing addictive behaviors toward screen media [30]. On average, 95% of US adolescents reported using YouTube, followed by TikTok (67% of US adolescents) and Instagram (62% of adolescents). More than half (54%) of US adolescents report it would be hard for them to give up social media [29].

Although this study is the first to use a US-based nationally representative sample to test differences in ST by developmental stage and periods of the day, it is not exempt of limitations. Thigh-worn accelerometers are more accurate than waist-worn accelerometers for measuring ST (due to their ability to assess participant posture). However, it has been reported that thigh-worn accelerometers reduce wear-time compliance [31, 32] due to skin irritation [31, 32] or having a sweaty thigh [31]. Thus, the hip-worn accelerometer provides a more comfortable placement, which increases adherence, while still providing a valid measure of ST when using validated cut-points. In our study, we used Evenson et al. cut-points [33], which have been validated for our age range. Second, our data were collected between 2003 and 2006, which raises concerns related to the relevance of findings in 2022, since societal ST patterns can change over time. Indeed, observational studies [12, 34] have shown increases in the quantity of ST with the easy accessibility to internet access. Dalene et al. [12] measured device-assessed ST in 5,168 Norwegian children and adolescents in years 2005, 2011, and 2018. The authors reported increases of weekly ST between 2005 and 2018 of 6% in 9-year-old boys, 1% in 9-year-old girls, and 4% in 15-year-old boys and girls [12]. Similarly, Schroeder et al. [34] collected ST data in US youth in 2017. They reported that children (8 – 12 years old) spent an average of 8.3 ± 2.1 h/day in ST. This suggests that in the decade between when NHANES distributed hip-worn accelerometers (2003–2006) and 2017, there may have been a ~ 5% increase in ST among US youth. Lastly, the present study does not fill the gap about *why* adolescents are more sedentary during each period of the day. Therefore, future studies should be conducted to gather information about the barriers and facilitators of ST in adolescence specific to each period of the weekday and weekend day. The design of the study also does not allow us to determine causality; therefore, we cannot say that too much ST is caused by being an adolescent, though the inverse (ST causes adolescence) is impossible.

## Conclusion

The current study’s analyses accounted for the NHANES complex sampling design to account for stratification, clustering, and weighting as well as sex, race/ethnicity, annual family income, and body mass index. Although previous cross-sectional and longitudinal studies have shown adolescents are more sedentary than children, to our knowledge, this is the first study to examine differences by periods of the day in a US sample. Findings confirm adolescents are more sedentary than children during most of the weekday periods (during school, afterschool, and the evening) and all weekend day periods (weekend mornings, afternoons, and evenings), though there is substantial variability in the magnitude of the disparities by period of the day (weekly disparities ranging from 9 to 35%). Evidence suggests that during school and afterschool periods contributed most to sedentary disparities overall, though substantial disparities also exist in periods of the day that correspond with time at home. This provides insight for future interventions, on when and where interventions should seek to reduce ST levels in adolescents.

## Supplementary Information

**Additional file 1: Table A1.** Accelerometer Data Processing Specifications [35, 36].

**Additional file 2: Table A2.** Description of the Linear Regression Syntax for SAS.

**Additional file 3: Table A3.** Regressions of Sedentary Time on Each Weekday Period on Developmental Stage.

**Additional file 4: Table A4.** Regressions of Sedentary Time During Each Weekend Period on Developmental Stage.

## Data Availability

The datasets used and/or analyzed during the current study are from the U.S. Department of Health & Human Services. The variable list as well as the data are available at the Centers for Disease Control and Prevention website: https://www.cdc.gov/nchs/nhanes/index.htm.

## Abbreviations

ST: Sedentary Time
NHANES: National Health and Nutrition Examination Survey
US: United States
