# Supplementary material for: Developmental disparities in sedentary time by period of the day among US youth: a cross-sectional study
Source: BMC Public Health. 2022 Nov 8;22:2047. doi: 10.1186/s12889-022-14447-4 (PMC9644603; doi:10.1186/s12889-022-14447-4)
Supplement: Supplementary file 1 — Additional file 1: Table A1. Accelerometer Data Processing Specifications [35, 36]. [file 12889_2022_14447_MOESM1_ESM.docx]

| **Table A1. Accelerometer Data Processing Specifications** | |
| --- | --- |
| **Features** | **Specifications** |
| Years | Both waves were selected (2003-2004 and 2005-2006) |
| Wear Time (35) | The simple algorithm was applied separately to each 24-hour period since participants were instructed to take off the device for sleep time and aquatic activities. The minimum length of non-wear period was set up to 60 minutes, tolerance to two minutes, and the maximum count value for minutes with non-zero counts were 99 counts. In this case, wear time was obtained by subtracting non-wear time from 24 hours. Overall, the algorithm classifies as non-wear periods any interval of at least 60 consecutive minutes of no activity intensity counts; however, since tolerance was set up to two minutes, it will allow one to two minutes of counts between 0 – 99 as wear time. |
| Artifacts | The cutpoint to flag counts as artifacts rather than meaningful measurements was 25000 counts. This function allows researchers to correct abnormally high count-values in the accelerometer data by substituting such values with the mean of the count values that are considered normal. |
| Compliance (36) | A valid day was defined as ≥ 600 min or 10 hours of wear time. A valid week was defined as 4 or more days of valid data, which included at least three valid weekdays and 1 weekend day. Hence, in order to be included participants needed to meet criteria for a valid week and valid weekend, which were operationalized as having sufficient valid days in each category. |
| Variables to Calculate | We selected the option that includes basic indicators of volume + intensity/bout variables + an hourly variable. The basic indicators of volume permit to obtain variables about participant ID, wave, day of the week, valid days, valid week, valid wear time, and counts per minutes. The intensity/bout variables include steps, minutes and counts in ST, percentage of the day in ST, ST bouts, and minutes and counts in each physical activity intensity. The hourly variables included minutes per hour of ST during each hour of the day.  We calculated averages (minutes per hour in ST) for weekdays and weekends separately. |
| Intensity Cutpoints | Sedentary time cut point was based on Evenson’s cut points (33) for children and adolescents (between 5 and 17 years), which define  ST ≤ 100 cpm. |
| Miscellaneous | No calculation of average cpm as average daily counts divided by average daily wear time was conducted. We selected sedentary minutes as the hourly activity variable to be recorded. Sixty minutes was the wear time per hour required to record a measurement or to calculate minutes per hour in ST. We did not normalize the hourly variable by wear time because the outcome already accounts for wear time (minutes per hour). |
| Other Data to Merge | Demographics were selected. |
| Notes. ST = sedentary time, cpm = counts per minute. | |
