# Supplementary material for: Developmental disparities in sedentary time by period of the day among US youth: a cross-sectional study
Source: BMC Public Health. 2022 Nov 8;22:2047. doi: 10.1186/s12889-022-14447-4 (PMC9644603; doi:10.1186/s12889-022-14447-4)
Supplement: Supplementary file 3 — Additional file 3: Table A3. Regressions of Sedentary Time on Each Weekday Period on Developmental Stage. [file 12889_2022_14447_MOESM3_ESM.docx]

| **Table A3. Regressions of Sedentary Time on Each Weekday Period on Developmental Stage** | | | | | |
| --- | --- | --- | --- | --- | --- |
| Weekday Periods | Variables | Unadjusted model  $R^{2}$ = 0.01 | | Adjusted model  $R^{2}$ = 0.02 | |
|  |  | b (95% CI) | P | b (95% CI) | P |
| Before School | Childhood | -2.7 (-4.8, -0.6) | 0.01 | - | - |
|  | Female | - | - | - | - |
|  | Non-Hispanic White | - | - | - | - |
|  | Non-Hispanic Black | - | - | **-** | **-** |
|  | Other Hispanic | - | - | - | - |
|  | Other Race – Including Multi Racial | - | - | - | - |
|  | Annual Family Income | - | - | - | - |
|  | Body Mass Index | - | - | - | - |
| During School |  | $R^{2}$ = 0.23 | | $R^{2}$ = 0.27 | |
|  | Childhood | **-8.2 (-8.9, -7.5)** | **<0.0001** | **-7.4 (-8.1, -6.6)** | **<0.0001** |
|  | Female | - | - | **2.8 (2.2, 3.5)** | **<0.0001** |
|  | Non-Hispanic White | - | - | -0.2 (-1.1, 0.7) | 0.59 |
|  | Non-Hispanic Black | - | - | -0.3 (-1.0, 0.3) | 0.33 |
|  | Other Hispanic | - | - | 0.6 (-1.0, 2.1) | 0.46 |
|  | Other Race – Including Multi Racial | - | - | 1.3 (-0.3, 3.0) | 0.11 |
|  | Annual Family Income | - | - | 0.3 (-0.8, 1.3) | 0.63 |
|  | Body Mass Index | - | - | **3.8 (2.3, 5.2)** | **<0.0001** |
| Afterschool |  | $R^{2}$ = 0.26 | | $R^{2}$ = 0.29 | |
|  | Childhood | **-8.9 (-9.7, -8.2)** | **<0.0001** | **-7.8 (-8.6, -7.0)** | **<0.0001** |
|  | Female | - | - | **2.1 (1.3, 2.9)** | **<0.0001** |
|  | Non-Hispanic White | - | - | 0.5 (-0.3, 1.2) | 0.19 |
|  | Non-Hispanic Black | - | - | **-1.2 (-1.9, -0.4)** | **0.003** |
|  | Other Hispanic | - | - | 0.4 (-1.3, 2.1) | 0.66 |
|  | Other Race – Including Multi Racial | - | - | 1.2 (-1.3, 2.1) | 0.31 |
|  | Annual Family Income | - | - | -0.2 (-1.1, 0.7) | 0.63 |
|  | Body Mass Index | - | - | **5.1 (3.0, 7.2)** | **<0.0001** |
| Evening |  | $R^{2}$ = 0.21 | | $R^{2}$ = 0.23 | |
|  | Childhood | **-8.4 (-8.1, -7.2)** | **<0.001** | **-7.6 (-8.5, -6.8)** | **<0.0001** |
|  | Female | - | - | **1.4 (0.5, 2.3)** | **0.004** |
|  | Non-Hispanic White | - | - | -0.4 (-1.3, 0.5) | 0.34 |
|  | Non-Hispanic Black | - | - | **-2.0 (-3.4, -0.7)** | **0.004** |
|  | Other Hispanic | - | - | 1.3 (-0.4, 3.0) | 0.13 |
|  | Other Race – Including Multi Racial | - | - | 0.5 (-1.8, 2.8) | 0.65 |
|  | Annual Family Income | - | - | -0.03 (-1.3, 1.2) | 0.96 |
|  | Body Mass Index | - | - | 3.8 (1.0, 6.5) | 0.008 |
