# Supplementary material for: Developmental disparities in sedentary time by period of the day among US youth: a cross-sectional study
Source: BMC Public Health. 2022 Nov 8;22:2047. doi: 10.1186/s12889-022-14447-4 (PMC9644603; doi:10.1186/s12889-022-14447-4)
Supplement: Supplementary file 4 — Additional file 4: Table A4. Regressions of Sedentary Time During Each Weekend Period on Developmental Stage. [file 12889_2022_14447_MOESM4_ESM.docx]

| **Table A4. Regressions of Sedentary Time During Each Weekend Period on Developmental Stage** | | | | | |
| --- | --- | --- | --- | --- | --- |
| Weekend Periods | Variables | Unadjusted Model  $R^{2}$ = 0.08 | | Adjusted Model  $R^{2}$ = 0.10 | |
|  |  | b (95% CI) | P | b (95% CI) | P |
| Morning | Childhood | **-7.7 (-9.6, -5.7)** | **<0.0001** | **-7.1 (-9.0, -5.1)** | **<0.0001** |
|  | Female | - | - | 0.6 (-0.7, 1.8) | 0.37 |
|  | Non-Hispanic White | - | - | 0.7 (-1.1, 2.4) | 0.43 |
|  | Non-Hispanic Black | - | - | **4.0 (2.2, 5.7)** | **<0.0001** |
|  | Other Hispanic | - | - | **4.5 (2.2, 6.9)** | **0.0005** |
|  | Other Race – Including Multi Racial | - | - | 2.0 (-2.3, 6.3) | 0.36 |
|  | Annual Family Income | - | - | 1.9 (-0.3, 4.1) | 0.08 |
|  | Body Mass Index | - | - | 3.3 (-0.2, 7.0) | 0.06 |
| Afternoon |  | $R^{2}$ = 0.25 | | $R^{2}$ = 0.27 | |
|  | Childhood | **-10.1 (-11.0, -9.1)** | **<0.0001** | **-8.9 (-9.9, -7.9)** | **<0.0001** |
|  | Female | - | - | **1.7 (0.9, 2.4)** | **0.0001** |
|  | Non-Hispanic White | - | - | 0.8 (-0.1, 1.7) | 0.08 |
|  | Non-Hispanic Black | - | - | 0.5 (-0.6, 1.7) | 0.34 |
|  | Other Hispanic | - | - | 1.7 (0.03, 3.3) | 0.045 |
|  | Other Race – Including Multi Racial | - | - | 2.8 (0.9, 4.8) | 0.01 |
|  | Annual Family Income | - | - | -0.2 (-1.3, 0.9) | 0.67 |
|  | Body Mass Index | - | - | **5.5 (3.1, 8.0)** | **<0.0001** |
| Evening |  | $R^{2}$ = 0.18 | | $R^{2}$ = 0.19 | |
|  | Childhood | **-9.1 (-10.2, -8.1)** | **<0.0001** | **-8.1 (-9.3, -7.0)** | **<0.0001** |
|  | Female | - | - | 1.0 (-0.0, 2.1) | 0.051 |
|  | Non-Hispanic White | - | - | 0.4 (-0.7, 1.6) | 0.44 |
|  | Non-Hispanic Black | - | - | -1.7 (-3.2, -0.08) | 0.04 |
|  | Other Hispanic | - | - | 0.8 (-1.5, 3.2) | 0.47 |
|  | Other Race – Including Multi Racial | - | - | 2.9 (0.8, 5.1) | 0.009 |
|  | Annual Family Income | - | - | -0.7 (-2.1, 0.7) | 0.32 |
|  | Body Mass Index | - | - | **5.0 (3.1, 6.8)** | **< 0.0001** |
